# Supplementary material for: Arabidopsis telomerase takes off by uncoupling enzyme activity from telomere length maintenance in space
Source: Nat Commun. 2023 Nov 29;14:7854. doi: 10.1038/s41467-023-41510-4 (PMC10686995; doi:10.1038/s41467-023-41510-4)
Supplement: Supplementary file 1 — Supplementary Information [file 41467_2023_41510_MOESM1_ESM.pdf]

Supplementary Figures

Supplementary Fig. 1

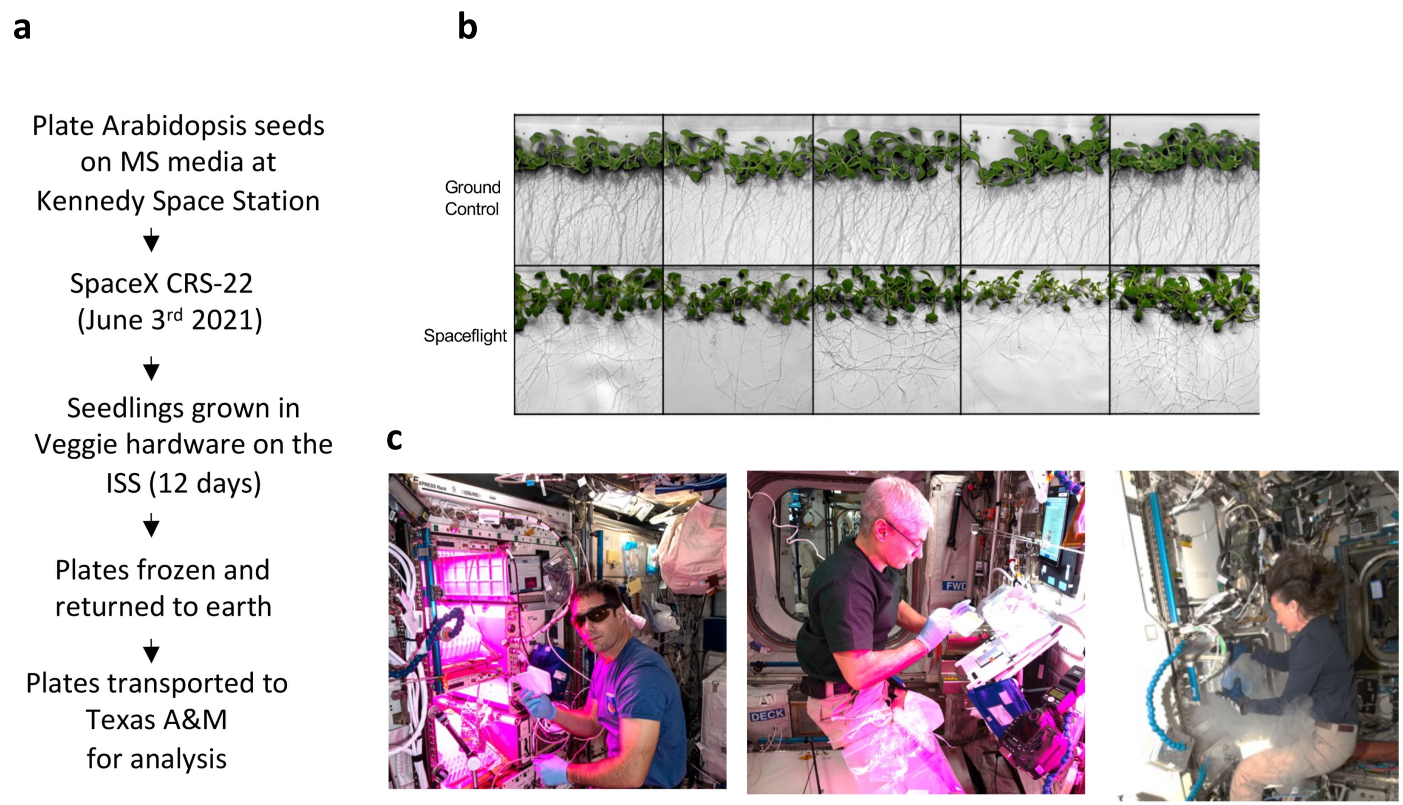

Supplementary Figure 1. Experimental design for space studies. **a** Experimental design and timeline of APEx-07. **b** Photo of *A. thaliana* seedlings grown on Veggie hardware as a Ground Control or on the ISS (spaceflight). Consistent with microgravity, roots from space-flown plants did not follow a gravitational growth vector. **c** Photos depicting Veggie hardware plate insertion (left), harvesting (middle) and cold storage (right) by astronauts aboard the ISS.

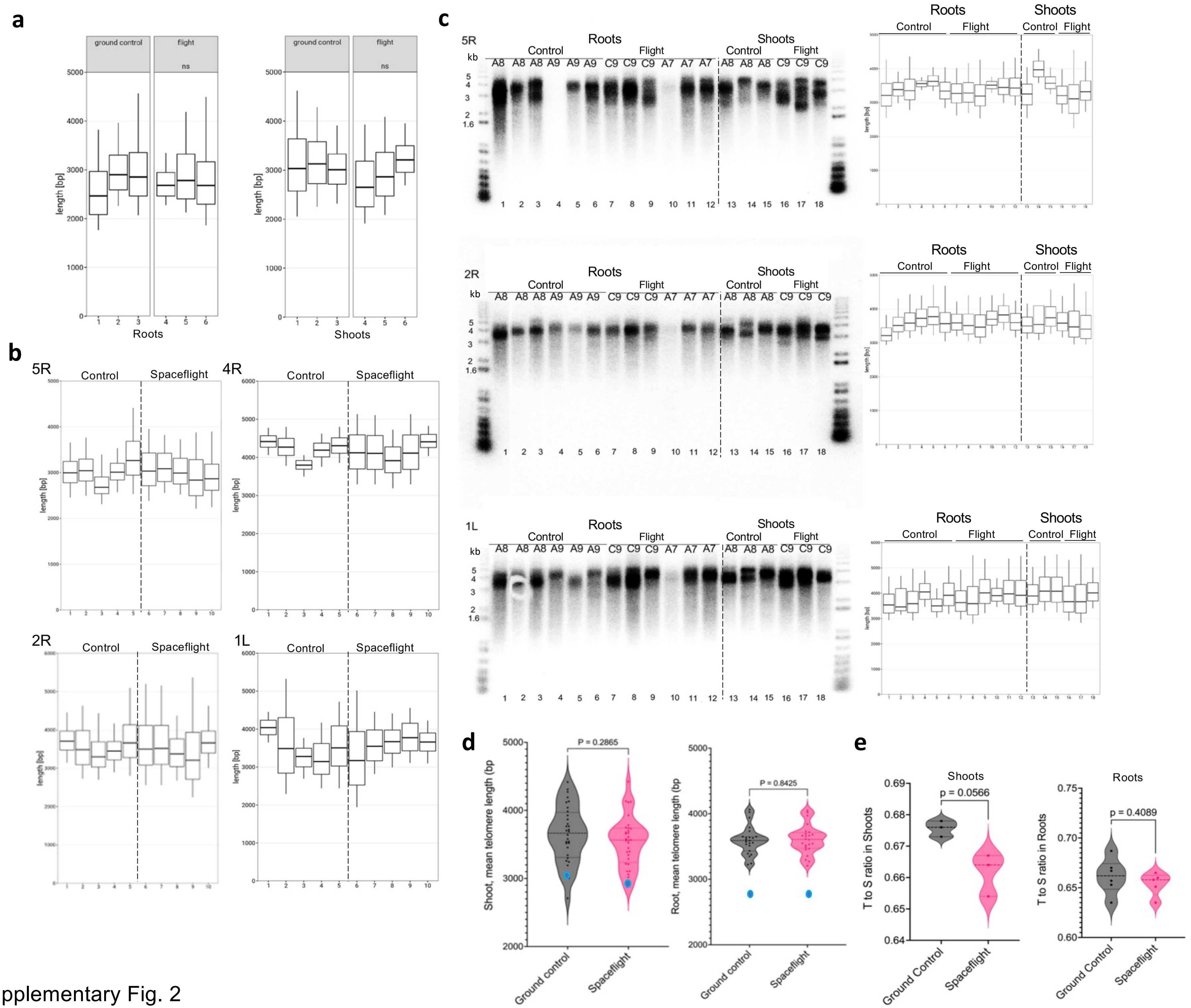

Supplementary Fig. 2

Supplementary Figure 2. Telomere length analysis for flight and ground control samples. a WALTER analysis of individual TRF lanes from Fig.1a, b for roots (left) and shoot (right). Lanes 1-3 are for ground controls (n = 3) and lanes 4-6 for spaceflight samples (n = 3). **b** WALTER analysis of shoot telomere length analyzed by PETRA lanes from Fig. 1c. Lanes 1-5 are ground control samples (n = 5) and lanes 6-10 for spaceflight samples (n = 5). Within the boxplot, the middle line represents the weighted median, box boundaries signify 25th and 75th percentiles, and extensions the lowest and highest values within the specific range of telomeres under consideration. **c** PETRA results and WALTER quantification for space-flown roots and shoots. (left) PETRA root data for 5R, 2R and 1L telomeres are the same as in Fig. 1d. N = 6 for both control and flight root samples. Additional shoot data are added to this figure (lanes 13-18). N = 3 for both control and flight shoot samples. These additional samples were used for quantification shown in Fig. 1e. (right) WALTER quantification for space-flown roots and shoots. Within the boxplot, the middle line represents the weighted median, box boundaries signify 25th and 75th percentiles, and extensions the lowest and highest values within the specific range of telomeres under consideration. Analysis shows no change in telomere length between root and shoot samples for either ground control or spaceflight. **d** (left) Combined mean telomere length of all chromosome arms for all biological shoot sample measured by PETRA and determined by WALTER. Blue dot represents means telomere length measured by TRF obtained from Fig 1a. Data shown as violin plots with p = 0.2865 by unpaired two-tailed Welch's t-test, $n_{control}$ = 32, $n_{spaceflight}$ = 32. (right) Combined mean telomere length of all chromosome arms for all biological root sample measured by PETRA and determined by WALTER. Blue dot represents means telomere length measured by TRF obtained from Fig. 1b. Data shown as violin plots with p = 0.8425 by unpaired two-tailed Welch's t-test, $n_{control}$ = 23, $n_{spaceflight}$ = 24. **e** (left)Telomere length analysis performed by T/S ratio of ground control (n = 3) and space-flown shoots (n = 3). Data shown as violin plots with p = 0.0566 measured by unpaired two-tailed Welch's t-test. (right) Telomere length analysis performed by T/S ratio of ground control (n = 6) and space-flown roots (n = 5). Data shown as violin plots with p = 0.4089 measured by unpaired two-tailed Welch's t-test. Source data provided as Source Data File.

Supplementary Fig.3

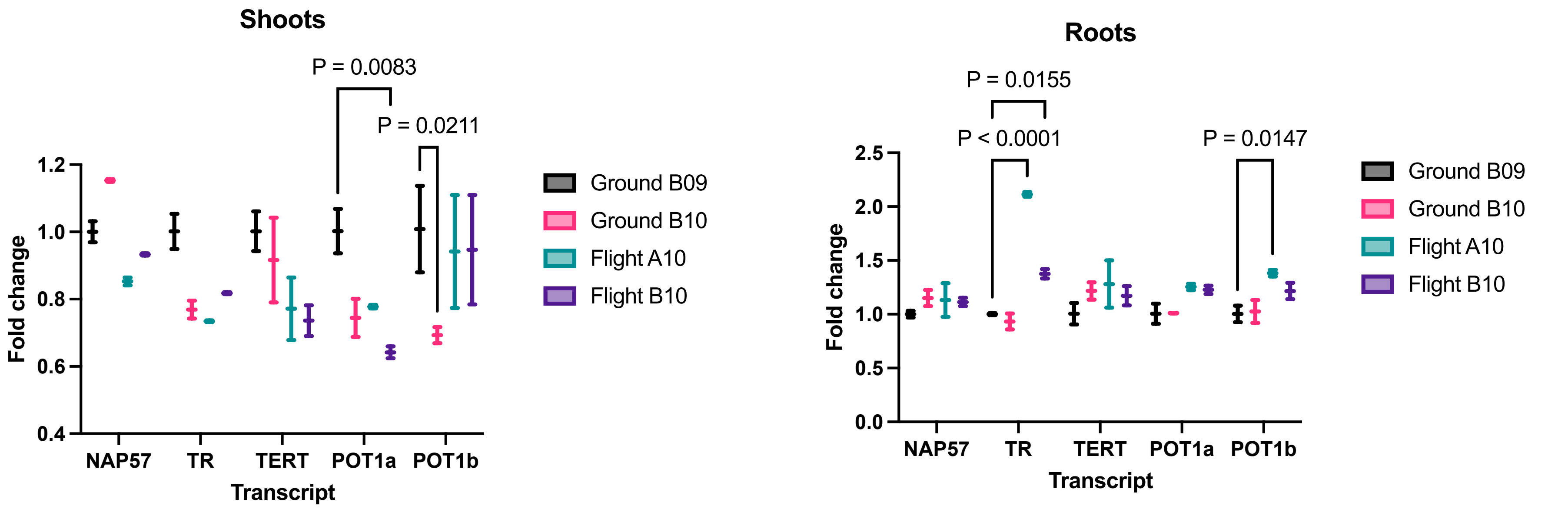

Supplementary Figure 3. Telomerase-associated gene expression. Two flight plates (A10, B10) and two ground plates (B09, B10) were dissected into shoot and root fractions and analyzed by qRT-PCR to assess expression of AtTERT, AtTR, dyskerin (NAP57), AtPOT1a and AtPOT1b genes using Actin2 as a reference. Data were normalized to ground sample B09. N = 2 for both flight and ground samples with 2 technical replicates for each sample. Data for each biological sample are shown with minima and maxima representing the technical replicates and the middle line representing the median of these two values. P calculated by two-way ANOVA. There is some variability between the two ground samples B09 and B10 leading to one of the ground samples being statistically significantly different to one of the flight samples. However, no consistent (between all samples) major fold changes were detected for any of the genes queried. Source data provided as Source Data File.

Supplementary Fig.4

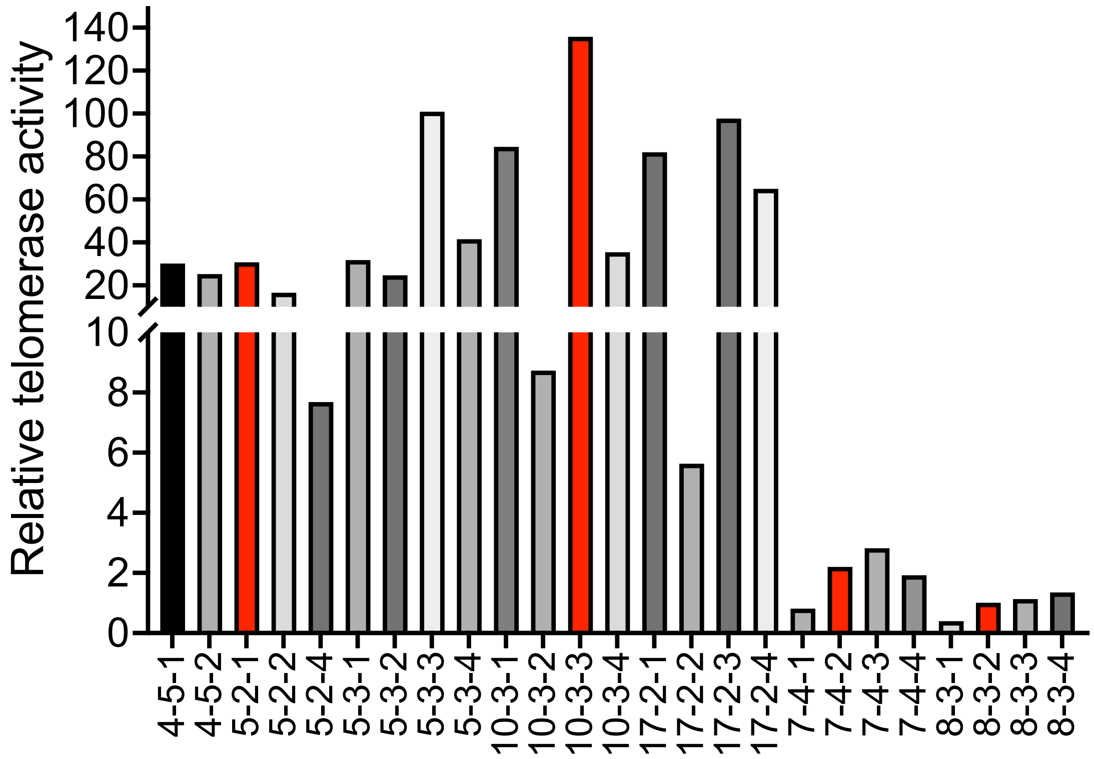

Supplementary Figure 4. Telomerase activity analysis in T3 Arabidopsis transgenic seedlings. Relative telomerase activity (measured by Q-TRAP) for independent T3 *A. thaliana* transformants carrying 35S:TERT or PTERT:TERT alleles in a *tert* mutant background. Source data provided as Source Data File.

Supplementary Fig.5

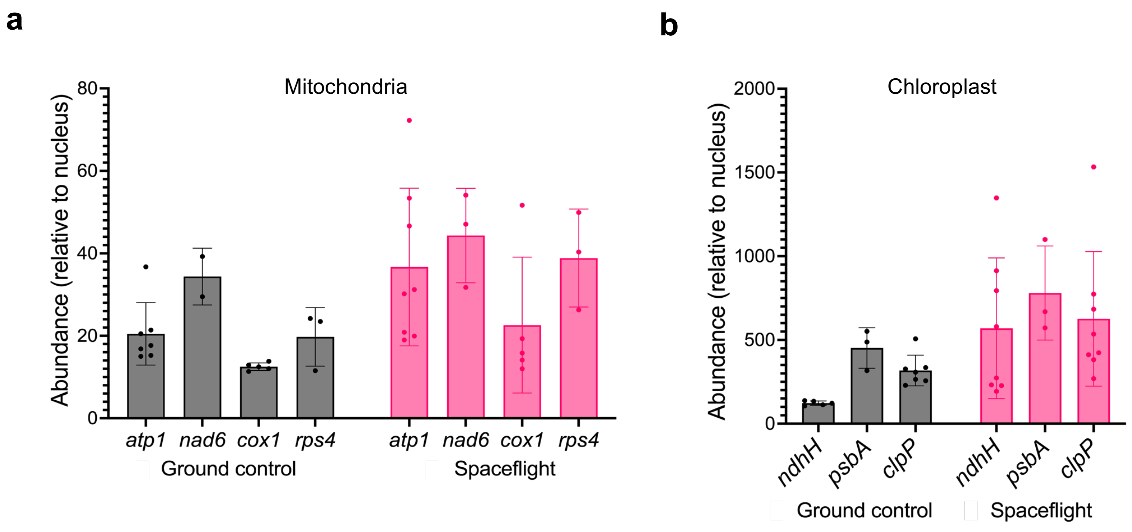

Supplementary Figure 5. Organellar DNA abundance assessed by qPCR of chloroplast and mitochondrial encoded genes. a mtDNA abundance relative to nuclear DNA was measured for ground control and spaceflight shoots (*n* = 15 for both) using the approach described in the Fig. 5b legend. Results represented as a boxplot displaying mean with *SD*. **b** cpDNA abundance relative to nuclear DNA measured using the approach described in the Fig. 5b legend for ground control and spaceflight shoots (*n* = 15 for both). Results represented as a boxplot displaying mean with *SD*. Source data provided as Source Data File.

Supplementary Fig. 6

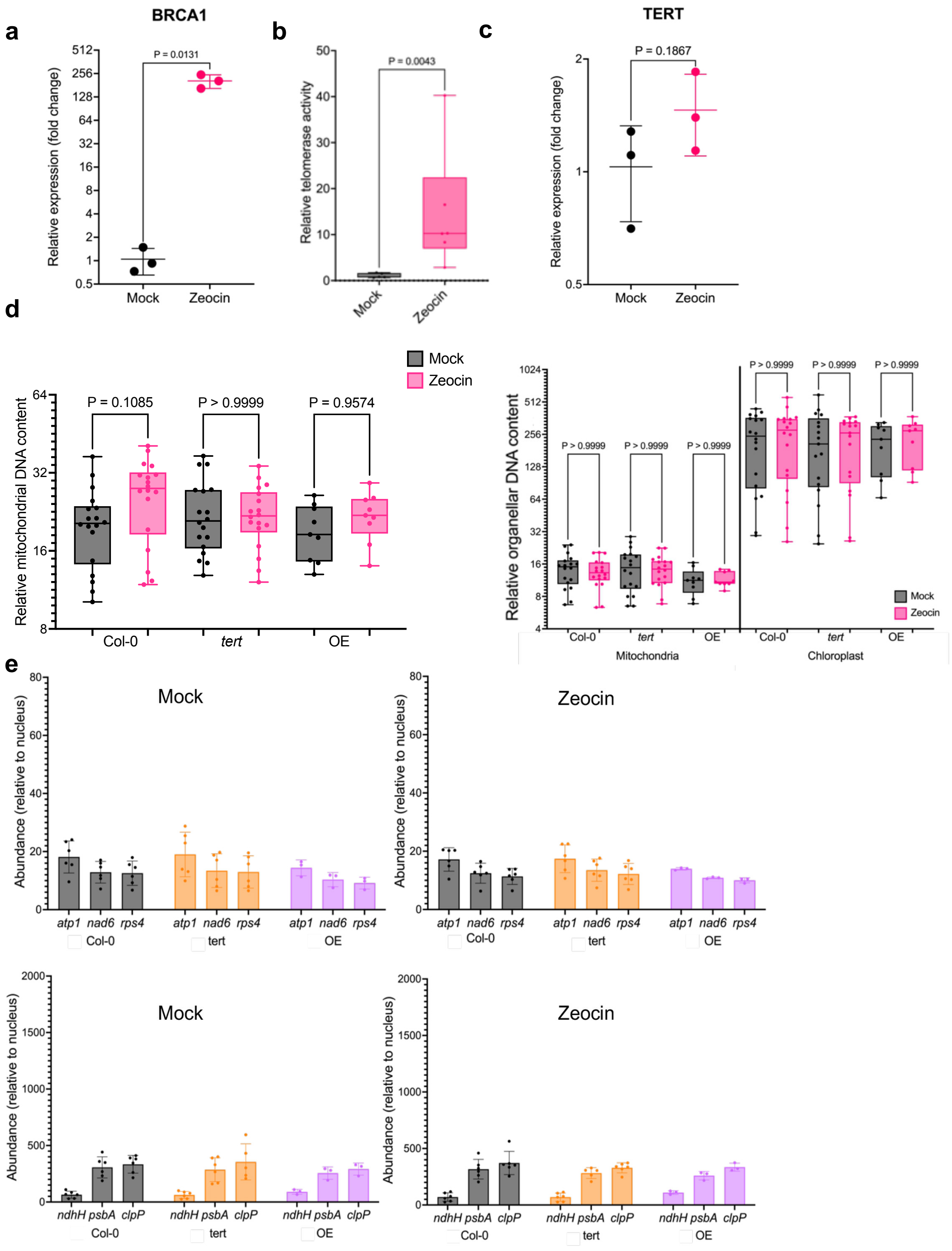

Supplementary Figure 6. Telomerase activity and organellar DNA in telomerase mutants and super-telomerase plants upon zeocin treatment. a qPCR of *BRCA1* transcripts in 12-day-old seedlings grown in 0.5 MS media with either mock or 20 μM zeocin treatment for 2 h (n = 3 for each treatment). Results represent mean with *SD*. P = 0.0131 by unpaired, two-tailed Welch's t-test. **b** Relative telomerase activity measured by Q-TRAP in mock (n = 5) and zeocin (n = 6) treated seedlings. For each biological replicate, 2 technical replicates were performed. Within the boxplot, the middle line represents the median, box boundaries signify 25th and 75th percentiles, and whiskers the lowest and highest values. Two-tailed Mann-Whitney test, p = 0.0043. **c** qPCR of *TERT* transcripts after zeocin treatment. N = 3 for both mock and zeocin treated samples. Results displayed as mean with *SD*. Unpaired two-tailed Welch's t-test, p = 0.1867. **d** Relative mtDNA in root (left), and mtDNA and cpDNA in shoots (right) measured by qPCR in mock and zeocin treated plants. N = 6 with 18 measurements per organelle. For organellar DNA abundance, 2 technical replicates were performed with each biological replicate for every organellar gene. Within the boxplot, the middle line represents the median, box boundaries signify 25th and 75th percentiles, and whiskers the lowest and highest values. P calculated by one way ANOVA. **e** cpDNA and mtDNA abundance relative to nuclear DNA measured using the approach described in the Fig. 5b legend for mock and zeocin treated samples (n = 6). Results represent mean with *SD*. Source data provided as Source Data File.

Supplementary Fig. 7

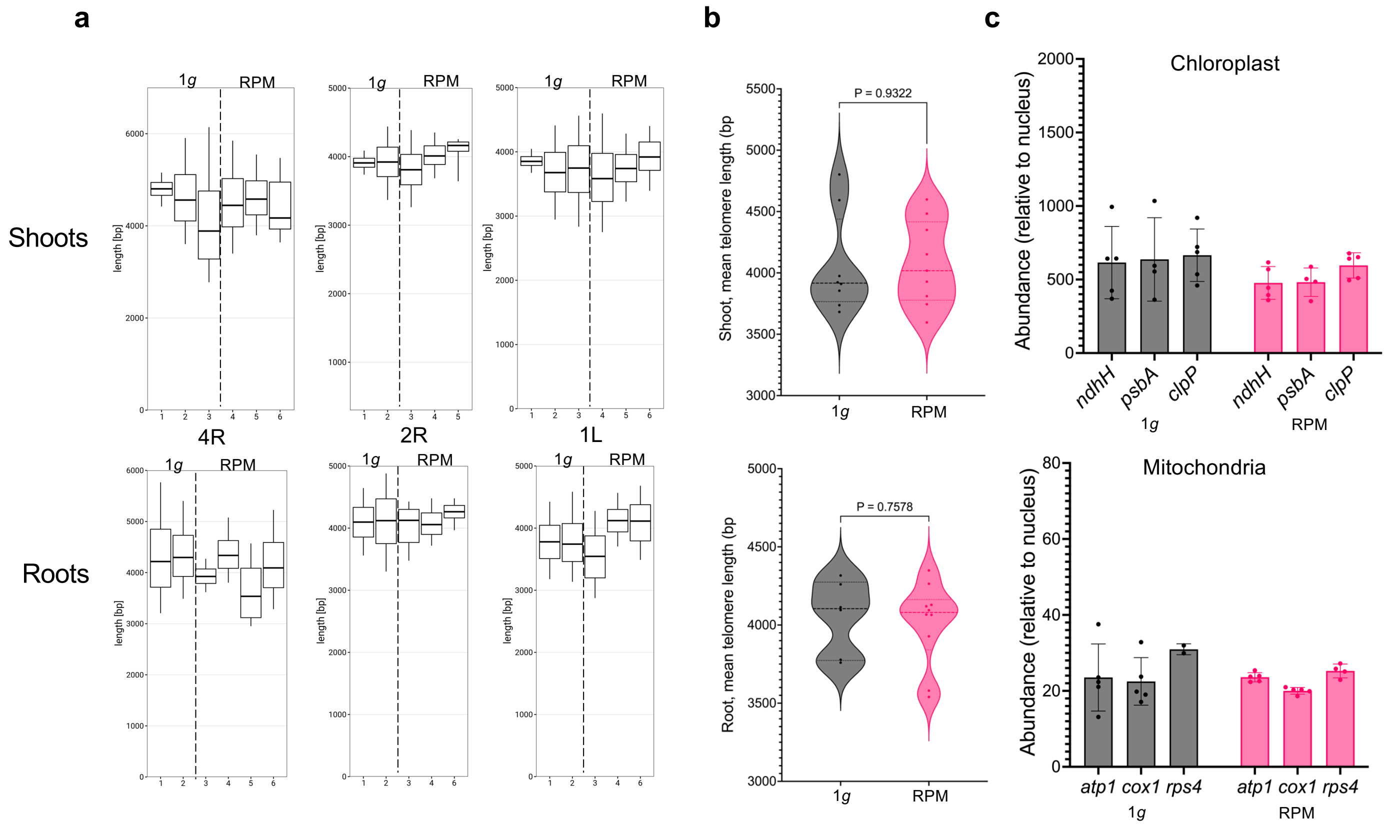

Supplementary Figure 7. Telomere length analysis of shoots and roots in RPM-grown plants. a WALTER analysis of individual PETRA lanes of shoot and root samples from Fig. 6b. Lane 1-3 are ground control (*n* = 3) and lane 4-6 are RPM samples (*n* = 3) for shoots. Lane 1-2 are control samples (*n* = 2) and 3-6 are RPM samples (*n* = 3) for roots. Within the boxplot, the middle line represents the weighted median, box boundaries signify 25th and 75th percentiles, and extensions the lowest and highest values within the specific range of telomeres under consideration. **b** Combined mean telomere length of all chromosome arms for all biological sample of shoot (top) and root (bottom) 35S::RPL18 samples as determined by WALTER. Results displayed as violin plot with all biological points. Shoot telomere length in 1*g* controls: 4060 bp; RPM shoots: 4076 bp (net difference 16 bp, $p = 0.9322$ by unpaired two-tailed Welch's *t*-test, $n_{1g} = 8$, $n_{\mathrm{RPM}} = 9$). Root 1*g*: 4054 bp; RPM: 4014 bp (net difference -40 bp, $p = 0.7578$ by unpaired two-tailed Welch's *t*-test, $n_{1g} = 6$, $n_{\mathrm{RPM}} = 10$). **c** mtDNA and cpDNA abundance relative to nuclear DNA for ground control and RPM shoot samples. For mitochondrial 1*g* (*n* = 13) and RPM (*n* = 14). For chloroplast 1*g* and RPM (*n* = 14). Results represent mean with *SD*. Source data provided as Source Data File.

Supplementary methods

DNA and protein extraction

Genomic DNA for telomere length, 8-oxoG, organellar qPCR studies was prepared from 6 pooled roots and 3 pooled shoots for each biological replicate. For DNA isolation, the cetyltrimethylammonium bromide (CTAB) method was performed[1]. Frozen plant DNA tissue was ground to a powder and mixed in a 1:1 ratio (w/v) with CTAB lysis buffer (100 mM Tris HCl pH 8.0, 20 mM EDTA pH 8.0, 1.4 M NaCl, 2% CTAB, 2% ß-mercaptoethanol) and incubated at 65°C for 1 h; then, by adding phenol:chloroform:isoamyl alcohol (25:24:1) to the plant extract in a 1:1 ratio, total DNA was extracted into the aqueous phase by careful mixing. Phases were separated by centrifugation at 7,500 x *g* for 20 min. The aqueous phase was transferred to a new tube and DNA was precipitated with sodium acetate and 2-propanol. DNA was pelleted by centrifugation at 17,000 x *g* for 20 min, washed with 70% ethanol and treated with RNase A.

Protein extraction for telomerase activity assays was prepared from 6 pooled roots and 3 pooled shoots for each biological replicate. Plant tissue was ground using liquid nitrogen and homogenized with buffer W[2], The sample was centrifuged at 14,000 x *g* for 15 min at 4°C. The supernatant was collected and mixed with 350 µl of 50% PEG solution. The mixture was incubated on a lab shaker for 30 min. After centrifuging the sample at 14,000 x *g* for 5 min, the supernatant was discarded, and the pellet was gently resuspended in 100 µl of Buffer W. The sample was then incubated on a lab shaker for an additional 30 min for thorough resuspension. Protein concentration was calculated using a Bradford Assay. The sample was rapidly frozen in liquid nitrogen and stored at -80°C for long-term preservation. Buffer W composition consisted of H_2O, 1M Tris-Acetate pH 7.5, 1M $MgCl_2$, 2M Potassium Glutamate, 0.5M EGTA, PVPP and Glycerol.

Organellar DNA abundance

Mitochondrial and chloroplast DNA were measured using qPCR[3]. For nuclear genes, *RpoTm*: Forward Primer (fw): AGCCTGTGCGTAATGCTATTCA, Reverse Primer (rv): GCCATCTTATCAGCCGGTAACT; *RpoTp*: Forward Primer (fw): TGGAAGCCGTCTGCTAGAACTA, Reverse Primer (rv): TGTCTGAATGCAGGTCGAAAC. For mitochondrial genes, *nad6*: Forward Primer (fw): AGGATGTATTCCGACGAAATGC, Reverse Primer (rv): CGTGAGTGGGTCAGTCGTCC; *atp1:* Forward Primer (fw): CTTAGAAAGAGCGGCTAAACGA, Reverse Primer (rv): GGGAATATAGGCCGATACGTCT; *rps4*: Forward Primer (fw): CCCATCACAGAGATGCACAGA, Reverse Primer (rv): GGAGACGAAGCGGAATAACGT; *cox1*: Forward Primer (fw): GCCATGATCAGTATTGGTGTCTT, Reverse Primer (rv): CTACGTCTAAGCCCACAGTAAACA. For chloroplast genes, *clpP*: Forward Primer (fw): TATGCAATTTGTGCGACCC, Reverse Primer (rv): TTGGTAATTGCTCCTCCGACT; *psbA*: Forward Primer (fw): CATCCGTTGATGAATGGCTAT, Reverse Primer (rv): AACTAAGTTCCCACTCACGACC; *ndhH*: Forward Primer (fw): CATACCGGTGGCAGCTTCGAA, Reverse Primer (rv): TCATCTGTTATGGCTCGGCCC. PCR conditions were as followed for SYBR Green PowerUp, with a 95°C for 2 min denaturation followed by a 40 cycle amplification with denaturation for 95°C for 15 sec, and annealing and extension for 60°C for 30 sec.

Gene expression studies

Four datasets (GLDS-38, GLDS-120, GLDS-218 and GLDS-427)[4–7] and Col-0 were retrieved from GeneLab for comparison of telomeric gene expression in previous flights and re-analyzed. Primers and low-quality sequences were removed from reads using Trim Galore! under the base parameters. Paired reads were aliened to the Columbia genome with araport11 annotations using STAR. Gene counts were normalized using the trimmed mean of M values (TMM) as calculated using limma voom. Differential expression was calculated using the Empirical Bayes method in edgeR.

For qPCR of telomere-associated genes, two flight plates and two ground plates were dissected into shoot and root fractions. Tissues were ground to a fine powder in liquid nitrogen and total RNA was isolated by a silica column-based purification kit (Norgen). RNA was DNase treated to remove any genomic contamination (DNAfree, Ambion). Sample quantities were normalized based on UV-Vis absorption at 260nm and cDNA was synthesized from DNase treated RNA by reverse transcription (GoScript, Promega). Primers for *AtNAP57, AtTR, AtTERT, AtPOT1a* and *AtPOT1b* were derived from [8,9]. qPCR was carried out using Real-Time PCR thermal cycler (Agilent Technologies) and data were analyzed by instrument software (Step One v2.2.2) in reference to Actin2. The static reference gene PP2A was included as an additional control in some assays. Data were normalized to Ground sample B09.

For light stress treatments, RNA extraction and qRT-PCR were conducted as described above, using *GAPDH* as a reference gene. Primers used were: *RD29b* (RD29b-Fw AGCAAGCAGAAGAACCAATCA, RD29b-Rvs CTTTGGATGCTCCCTTCTCA), GAPDH (GAPDH-Fw ATGGCTGACAAGAAGATCAGAA, GAPDH-Rvs TTAGGCCTTTGACATGTGAAC).

For zeocin treatments total RNAs were isolated from frozen samples using TRIzol Reagent (Ambion). To remove residual genomic DNA from each preparation, total RNA was treated with DNase I (Invitrogen) and reverse transcription was conducted using SuperScript III RT (Invitrogen) according to the supplier's instructions. The levels of TERT and BRCA1 in Col-0 and *tert* G2 mutants were determined using 50 ng of RNA. qPCR was performed using a CFX Connect quantitative PCR apparatus (Bio-Rad). PowerUp SYBR green (Applied Biosystems) master mix was used for qPCR. Each result was analyzed using CFX Maestro software (Bio-Rad). *Actin2* (*ACT2*) was used as an internal control. Primers were: Actin2 (Forward: CAGCCCTCGTTTGTGGGAAT, Reverse: GGTTCCACCACTGAGCACAA), BRCA1 (Forward: TGCATCCATTAAGTTGCCCTGTG, Reverse: TAGGCTGAGAGTGCAGTGGTTC).

Supplementary references

1. Doyle, J. J. D. A rapid DNA isolation procedure for small quantities of fresh leaf tissue. *Phytochem. Bull.* **19**, 11–15 (1987).
2. Kannan, K., Nelson, A. D. L. & Shippen, D. E. Dyskerin Is a component of the Arabidopsis telomerase RNP required for telomere maintenance. *Mol. Cell. Biol.* **28**, 2332–2341 (2008).
3. Weihe, A. Quantification of organellar DNA and RNA using real-time PCR. *Methods Mol. Biol.* **1132**, 235–243 (2014).
4. Kruse, C. P. S. *et al.* Spaceflight induces novel regulatory responses in Arabidopsis seedling as revealed by combined proteomic and transcriptomic analyses. *BMC Plant Biol.* **20**, (2020).
5. Paul, A.-L. *et al.* Genetic dissection of the Arabidopsis spaceflight transcriptome: Are some responses dispensable for the physiological adaptation of plants to spaceflight? *PLoS One* **12**, e0180186 (2017).
6. Zhou, M., Sng, N. J., Lefrois, C. E., Paul, A. L. & Ferl, R. J. Epigenomics in an extraterrestrial environment: organ-specific alteration of DNA methylation and gene expression elicited by spaceflight in *Arabidopsis thaliana*. *BMC Genomics 2019 201* **20**, 1–17 (2019).
7. Paul, A. L., Haveman, N., Califar, B. & Ferl, R. J. Epigenomic Regulators Elongator Complex Subunit 2 and Methyltransferase 1 differentially condition the spaceflight response in Arabidopsis. *Front. Plant Sci.* **12**, 1907 (2021).
8. Bose, S. *et al.* tRNA ADENOSINE DEAMINASE 3 is required for telomere maintenance in *Arabidopsis thaliana*. *Plant Cell Rep.* **39**, 1669–1685 (2020).

9. Song, J. *et al.* The conserved structure of plant telomerase RNA provides the missing link for an evolutionary pathway from ciliates to humans. *Proc. Natl. Acad. Sci. U. S. A.* **116**, 24542–24550 (2019).
