# Supplementary material for: Arabidopsis telomerase takes off by uncoupling enzyme activity from telomere length maintenance in space
Source: Nat Commun. 2023 Nov 29;14:7854. doi: 10.1038/s41467-023-41510-4 (PMC10686995; doi:10.1038/s41467-023-41510-4)
Supplement: Supplementary file 3 — Reporting Summary [file 41467_2023_41510_MOESM3_ESM.pdf]

nature portfolio

Corresponding author(s): Dr. Dorothy Shippen
Dr. Sarah Wyatt

Last updated by author(s): 8.15.23

Reporting Summary

Nature Portfolio wishes to improve the reproducibility of the work that we publish. This form provides structure for consistency and transparency in reporting. For further information on Nature Portfolio policies, see our Editorial Policies and the Editorial Policy Checklist.

Statistics

For all statistical analyses, confirm that the following items are present in the figure legend, table legend, main text, or Methods section.

n/a	Confirmed	
☐	☒	The exact sample size (*n*) for each experimental group/condition, given as a discrete number and unit of measurement
☐	☒	A statement on whether measurements were taken from distinct samples or whether the same sample was measured repeatedly
☐	☒	The statistical test(s) used AND whether they are one- or two-sided<br>*Only common tests should be described solely by name; describe more complex techniques in the Methods section.*
☐	☒	A description of all covariates tested
☐	☒	A description of any assumptions or corrections, such as tests of normality and adjustment for multiple comparisons
☐	☒	A full description of the statistical parameters including central tendency (e.g. means) or other basic estimates (e.g. regression coefficient) AND variation (e.g. standard deviation) or associated estimates of uncertainty (e.g. confidence intervals)
☐	☒	For null hypothesis testing, the test statistic (e.g. F, t, r) with confidence intervals, effect sizes, degrees of freedom and P value noted<br>*Give P values as exact values whenever suitable.*
☐	☒	For Bayesian analysis, information on the choice of priors and Markov chain Monte Carlo settings
☐	☒	For hierarchical and complex designs, identification of the appropriate level for tests and full reporting of outcomes
☒	☐	Estimates of effect sizes (e.g. Cohen's *d*, Pearson's *r*), indicating how they were calculated

Our web collection on statistics for biologists contains articles on many of the points above.

Software and code

Policy information about availability of computer code

Data collection	GE Typhoon FLA 9500, BIO RAD CFX96, BIO RAD C1000 Touch Thermal Cycler, BioTek EPOCH Serial#1211289, Thermo Scientific Genesys 10S VIS
Data analysis	For telomeric gene expression studies, primers and low-quality sequences were removed from reads using Trim Galore! under the base parameters. Paired reads were aligned to araport11 using STAR. Gene counts were normalized using the trimmed mean of M values (TMM) as calculated using limma voom. Differential expression was calculated using the Empirical Bayes method in edgeR. Trim Galore! version 0.6 under the base parameters. STAR version 2.6 and the araport11 genome annotations. limma voom version = 3.52. edgeR version = 3.38<br>Telomere length was analyzed using WALTER toolset v2.0<br>Q-PCR of telomeric genes was analyzed by Agilent Technologies software (Step One v2.2.2)<br>Q-TRAP was analyzed by using BioRad CFX Manager 3.1<br>PRISM 9 Version 9.5.1 (528), January 24, 2023

For manuscripts utilizing custom algorithms or software that are central to the research but not yet described in published literature, software must be made available to editors and reviewers. We strongly encourage code deposition in a community repository (e.g. GitHub). See the Nature Portfolio guidelines for submitting code & software for further information.

Data

Policy information about availability of data

All manuscripts must include a data availability statement. This statement should provide the following information, where applicable:

- Accession codes, unique identifiers, or web links for publicly available datasets
- A description of any restrictions on data availability
- For clinical datasets or third party data, please ensure that the statement adheres to our policy

Datasets analyzed for telomeric gene analysis from previous flights were retrieved from NASA's Genelab general data repository, which included GLDS-3845, GLDS-12047, GLDS-21846 and GLDS-42748. In addition datasets generated during and/or analyzed during the current study are available from the corresponding authors upon reasonable request. Source data are provided as a Source Data file.

Research involving human participants, their data, or biological material

Policy information about studies with human participants or human data. See also policy information about sex, gender (identity/presentation), and sexual orientation and race, ethnicity and racism.

Reporting on sex and gender	N/A
Reporting on race, ethnicity, or other socially relevant groupings	N/A
Population characteristics	N/A
Recruitment	N/A
Ethics oversight	N/A

Note that full information on the approval of the study protocol must also be provided in the manuscript.

Field-specific reporting

Please select the one below that is the best fit for your research. If you are not sure, read the appropriate sections before making your selection.

☒ Life sciences ☐ Behavioural & social sciences ☐ Ecological, evolutionary & environmental sciences

For a reference copy of the document with all sections, see nature.com/documents/nr-reporting-summary-flat.pdf

Life sciences study design

All studies must disclose on these points even when the disclosure is negative.

Sample size	Sample size was determined without pre-calculations. All experiments were simultaneously performed on at least three biological samples (with the exception of TRAP for space-flown roots and gene expression analysis for space-flown roots and shoots as material was limited).
Data exclusions	No data was excluded
Replication	All provided data underwent independent replication at least once more. In the case of all PCR- and ELISA-based assays there was a minimum of 3 technical replicates.
Randomization	No randomization was performed for this study.
Blinding	Blinding was omitted in the study due to specific design considerations or practical constraints.

Reporting for specific materials, systems and methods

We require information from authors about some types of materials, experimental systems and methods used in many studies. Here, indicate whether each material, system or method listed is relevant to your study. If you are not sure if a list item applies to your research, read the appropriate section before selecting a response.

Materials & experimental systems

n/a	Involved in the study	
☐	☒	Antibodies
☒	☐	Eukaryotic cell lines
☒	☐	Palaeontology and archaeology
☒	☐	Animals and other organisms
☒	☐	Clinical data
☒	☐	Dual use research of concern
☐	☒	Plants

Methods

n/a	Involved in the study	
☒	☐	ChIP-seq
☒	☐	Flow cytometry
☒	☐	MRI-based neuroimaging

Antibodies

Antibodies used	8-oxoG (Invitrogen™, ThermoFisher Scientific Cat. # EIADNAD).
Validation	Commercially available antibody. Validation statement on the manufacturer's website; "Rigorous validation<br><br>Each manufactured lot of this ELISA kit is quality tested for criteria such as sensitivity, specificity, precision, and lot-to-lot consistency. See manual for more information on validation."

Dual use research of concern

Policy information about dual use research of concern

Hazards

Could the accidental, deliberate or reckless misuse of agents or technologies generated in the work, or the application of information presented in the manuscript, pose a threat to:

No	Yes	
☒	☐	Public health
☒	☐	National security
☒	☐	Crops and/or livestock
☒	☐	Ecosystems
☒	☐	Any other significant area

Experiments of concern

Does the work involve any of these experiments of concern:

No	Yes	
☒	☐	Demonstrate how to render a vaccine ineffective
☒	☐	Confer resistance to therapeutically useful antibiotics or antiviral agents
☒	☐	Enhance the virulence of a pathogen or render a nonpathogen virulent
☒	☐	Increase transmissibility of a pathogen
☒	☐	Alter the host range of a pathogen
☒	☐	Enable evasion of diagnostic/detection modalities
☒	☐	Enable the weaponization of a biological agent or toxin
☒	☐	Any other potentially harmful combination of experiments and agents
